# The effect of transdermal nitroglycerin on pain control in diabetic patients with peripheral neuropathy

**DOI:** 10.1186/s40200-015-0217-3

**Published:** 2015-12-01

**Authors:** Arman Taheri, Arash Farbood, Ramin Heshmat, Afshin Samadi, Patricia Khashayar, Mostafa Qorbani, Mohammad Ghorbani, Ghazaleh Ebrahimi Khaneqah

**Affiliations:** Department of Anesthesiology, Amir Alam Hospital, Tehran University of Medical Sciences, Tehran, Iran; Shiraz Anesthesiology and Critical Care Research Center, Shiraz University of Medical Sciences, Shiraz, Iran; Endocrinology and Metabolism Research Institute, Chronic Diseases Research Center, Tehran University of Medical Sciences, Tehran, Iran; Osteoporosis Research Center, Tehran University of Medical Sciences, Tehran, Iran; Department of Community Medicine, School of Medicine, Alborz University of Medical Sciences, Karaj, Iran; Emergency Disaster Center of Ministry of Health and Medical Education, Tehran, Iran; Department: Anesthesiology and Critical Care Medicine, Institution: Shiraz University of Medical Sciences, Shiraz, Iran; Department of Anesthesiology, Shahid Faghihi Hospital, Zand Boulevard, Shiraz, Fars Iran

**Keywords:** Diabetes complications, Painful polyneuropathy, Nitroglycerin

## Abstract

**Background:**

Despite high prevalence of diabetic peripheral neuropathy there is no definite treatment for the condition. The present study was conducted to assess the efficacy of transdermal nitroglycerin patch in pain control of patients with DPN.

**Methods:**

This randomized, double-blind, crossover study was conducted on 30 patients with symmetric distal peripheral neuropathy and good glycemic control. The patients were randomly assigned to receive nitroglycerin transdermal and placebo patches in two 4-week stages. The severity of pain and other neuropathic sensory symptoms were assessed at the end of each course.

**Results:**

Mean reduction of pain severity was more prominent in the NTG group compared to placebo group of the study (*p* = 0.048) at least during the first phase of the study. Except for mood and sleep, a significant reduction in all Brief Pain Inventory scores was noted in the drug group (all corrected *p* < 0.05). SF-MPQ also showed the drug patch to be effective in improving different aspects of pain measured using McGill Pain Questionnaire, except for Role–emotional.

**Conclusions:**

It could be concluded that nitroglycerin plasters can effectively help alleviate pain in patients with diabetic neuropathy.

**Trial registration:**

IRCT201308223213N1

## Background

About 220 million individuals suffered from diabetes in 2010; the rate is expected to increase worldwide and reach 366 million in 2030 [1]. Peripheral neuropathy is one of the most common complications in patients with long-standing diabetes, and as many as 50 % of patients develop neuropathy by 25 years after diagnosis [2, 3]. Ten percent of the diabetic patients experience pain in association with their diabetic peripheral neuropathy (DPN).

The condition, mostly characterized by distal symmetrical symptoms and signs of sensory and/or motor impairment, is frequently underreported (12.5 %) and more frequently undertreated (39 %) [4]. DPN represents a diffuse duration-dependent injury to peripheral nerves that has major implications on quality of life, morbidity, and costs [5, 6]. The pain associated with diabetic peripheral neuropathy is a major cause of morbidity in these patients and may have a profound impact on their functioning and well-being.

There are several patterns of peripheral neuropathy based on the cause or size of predominant fiber involvement. The most common form is symmetrical peripheral neuropathy. Despite its high prevalence, there is no definite treatment for the condition. Several controlled studies have demonstrated that painful DPN can be relieved by antidepressants, anticonvulsants, tramadol, opioids, topical medications (analgesic patches, anesthetic patches, capsaicin cream, clonidine), aldose reductase inhibitors, and protein kinase C beta inhibitors [7–9]. Unfortunately, the use of these agents is often limited by the extent of pain relief provided and the occurrence of significant side effects. Thus, new compounds are needed in this regard.

The pathophysiology of the condition remains unclear, although it is associated with peripheral demyelination, a reduction in peripheral nerve conduction, and degeneration of myelinated and unmyelinated sensory fibers [10]. In many cases, ischemia and infarction in peripheral nervous system is responsible for the reported symptoms. Endoneurial capillaries are prone to ischemia. Based on the polyol pathway theory, the activation of this cascade results in oxidative stress and reduced nitric oxide (NO) levels. At the same time, it causes the formation of glycosylation end products, activation of hexosamine pathway, establishment of protein kinase C and finally certain changes in the neurotrophic factors, combination of which may lead to neuropathy [2, 11].

Recent data suggest that impaired neuronal generation plays an important role in the pathogenesis of DPN either through inducing hyperalgesia or contributing to a reduction in endoneurial blood flow in diabetic patients with peripheral sensory neuropathy [12–14]. In this regard and considering the fact that several studies have demonstrated that topical nitroglycerin can produce local vasodilation in the feet [15, 16], Yuen et al. found that isosorbide dinitrate spray (ISDN), a NO donor with potent local vasodilator properties, relieves some sensory symptoms, particularly pain and burning sensation, in a small number of diabetic patients [17].

In view of the fact that the ISDN spray is not available worldwide, Rayman et al. used glyceryltrinitrate (GTN) patches and showed it to be effective in reducing neuropathic pain in diabetic patients in a non placebo-controlled study [18]. The present study was therefore conducted to assess the efficacy of transdermal nitroglycerin patch in pain control of patients with DPN.

## Methods

This randomized, double-blind, crossover study was approved by the Ethical Board Committee of Diabetes Research Center of Tehran University of Medical Sciences (registration number: 00185). Eligible subjects included adult type 1 and type 2 diabetic patients with stable diabetic control not on any other medications for their neuropathic pain, visiting diabetes clinics of the Endocrinology and Metabolism Research Institute between May and September 2012. All the patients signed an informed consent form.

The patients were selected from among those referred to our diabetes clinic with symmetric distal peripheral neuropathy. None of the patients showed any indication that they had central, nociceptive, or psychogenic pain. Patients’ demographic data along with information on their type and duration of diabetes, time of onset of diabetic neuropathy were recorded in a questionnaire. Patients with erratic glycemic control defined as HbA1c levels higher than 8.5 % (69 mmol/mol), positive history of ischemic heart disease, peripheral arterial disease with absent foot pulses, active diabetic foot ulceration in the legs or a positive history of lower extremity amputation, and peripheral neuropathy secondary to other diseases such as hypothyroidism, vitamin B12 or folate deficiency, sarcoidosis and alcoholism were excluded. Subjects on concurrent vasodilator (sildenafil or nitroglycerin) therapy, those who had started taking insulin in the past month, or consumed medications with approved interaction with nitroglycerin were also excluded. Patients with pressure index ratio of ankle systolic pressure (mean of posterior tibial and dorsalis pedis) to brachial systolic pressure values lower than 0.8, which signified peripheral arterial disease as the cause of pain, were also excluded.

Upon arrival, a thorough neurologic physical examination and neurothesiometeric assessment were performed to confirm the presence of diabetic neuropathy (“The Neurothesiometer is used for the determination of vibration sensitivity threshold at any desired site on the body surface. Sensitivity decreases naturally with age but a number of medical conditions can be related to abnormal deterioration.”-http://www.algeos.com/neurothesiometer.html)

Peripheral neuropathy was diagnosed based on the presence of at least two of the following symptoms and signs: distal sensory impairment (touch, vibration, proprioception, pain), and distal bilateral muscle weakness or atrophy, bilateral decrease, or loss of tendon reflexes. In all cases, the diagnosis was verified by nerve conduction velocity, electromyography, and/or quantitative sensory tests.

Fasting venous blood samples were collected for the measurement of HbA1c at baseline and at the end of the study. Thyroid and liver function tests were performed at baseline to exclude other causes of neuropathy.

Considering the power of the study as 80 % (type II error = 0.2), remission rate of 44 % in the case group compared to 5 % in the controls, 23 patients were needed in each group. The patients were randomly assigned to start either in the drug or in the placebo limb of the study and after a washout period they were crossed over for receiving the treatment of the other group. Thereafter, taking into consideration the crossover nature of the study and possible 20 % loss to follow up rate, 30 patients were recruited in the study.

### Design of the drug trial

The patients were randomly assigned to receive nitroglycerin transdermal patch (Nitro-Dur 0.2 mg/h, Schering-Plough Pty Ltd., Australia) and placebo patches in two stages based on the odd and even number of their unit numbers. The patches were recommended to be applied once a day, worn for 12 to 14 h, and then removed. The patients were recommended to apply the patches to clean, dry and hairless skin, and choose a different area each day based on a cyclic pattern (right arm, right pectoral area, left pectoral area and left arm).

The patients were randomized to start the study either with drug (DP) or placebo (PD). Based on the groupings, the first phase of the treatment was started. After 4 weeks, there was a 3 week washout period during which the patients of either group were asked to taper the treatment by using the patches every other night at the first week and stop application of the patches for the next two weeks. Thereafter, the patients exchanged their treatment for a further 4 weeks. The patients and investigators were unable to distinguish between the patches as they were identical. No major changes in diabetic or pain management regimen were made before and throughout the study.

### Outcome measures

The severity of pain was assessed using 10-score Numerical Rating Scale (NRS) biweekly, where 0-no pain at all and 10-the most severe pain ever experienced. Those with NRS scores lower than four at baseline were excluded. The treatment efficacy was defined as 30 % and 50 % difference between the final score and the baseline score in NRS scoring for each treatment phase. Allodynia was assessed with suprathreshold testing method by using one of the largest Semmes-Wienstein monofilaments (6.10) and asking the patients to report the severity of pain according to a 3 point scale of mild, moderate and severe. Other neuropathic sensory symptoms (hot/cold sensation, tingling, numbness, hyperesthesia, jabbing-like sensation, and burning pain) were also recorded as no symptom: 0, mild: 1, moderate: 2, or severe: 3 on a 3-cm Likert scale.

At the end of each treatment phase, functional health and pain were assessed using SF-36 Health Survey and Brief Pain Inventory questionnaire (BPI), respectively. Beck Depression Inventory Score System (BDI) and standard seven-point Patients’ Global Impression of Change (PGIC) scale helped with depression and satisfaction evaluation at the same intervals.

The study assistant, an expert nurse unaware of the study objectives, called the patients twice a week and asked them about possible occurrence of pain and other adverse effects, its severity and duration at the end of each phase. Patients experiencing severe headache as a complication of nitroglycerin were excluded. Using a questionnaire, the patients were asked to report the influence of these adverse effects on their life. The study assistant also asked the patients regarding their satisfaction about the treatment, possible confounding use of analgesics or other rescue drugs. The patients were asked to visit the clinic at the end of each phase, during which he/she underwent a thorough physical examination.

### Statistical analysis

Continuous variables are expressed as mean (SD) when the distribution was normal or median (interquartile range) in the case lack of normal distribution. Categorical data are expressed as percentage. The normal distribution of continuous data was assessed by - Lilliefors’ test and due to lack of normality comparison of mean differences of the Beck Depression Inventory, Brief Pain Inventory and SF-MPQ parameters between drug and placebo groups were assessed by Wilcoxon Signed Rank test. Repeated measure ANOVA was used to compare the NRS at different intervals. The percent of patients’ allodynia and percent of PGIC reduction in study groups were assessed using Chi-square test. The gathered data were analyzed by SPSS ver. 15. A *p*-value less than 0.05 was considered statistically significant.

## Results

**Fig. 1:**
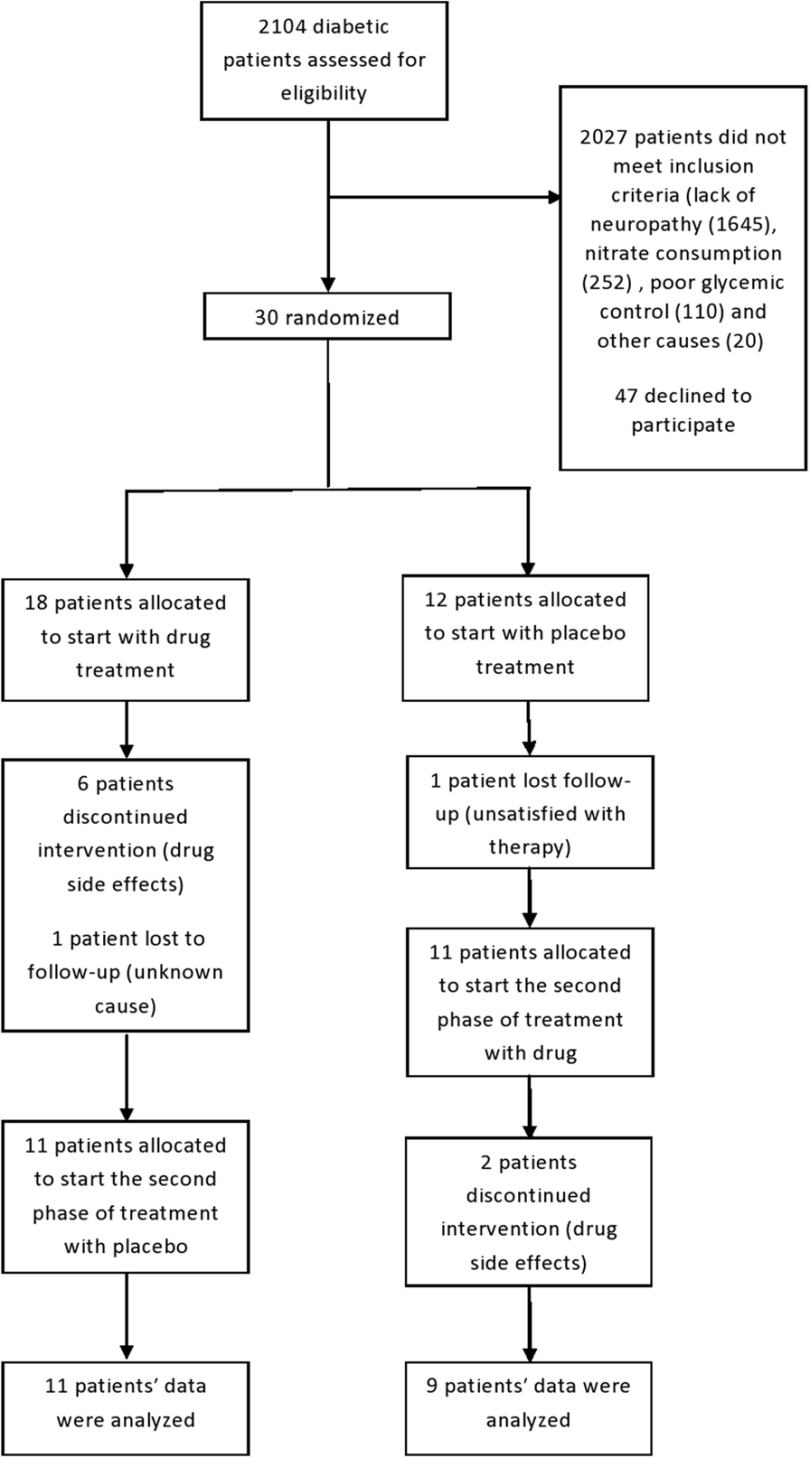
CONSORT flow-chart of the study

From among the 30 individuals recruited in the study, 10 did not complete the research, eight of them because of the adverse effects of the drug. Two others however, left as they were not willing to continue (Fig. 1). Table 1 outlines the adverse effects reported in these eight individuals. Sixteen females and four males with the mean age of 58.1 ± 10.9 years completed the study. Table 2 shows the demographic data of the participants.

**Table 1:** Drug side effects reported in the patients who left the study

	Cause of withdrawal	Severity
Case1	Headache	Moderate
	Vertigo, lightheadedness	Moderate
	Nausea, vomiting	Moderate
Case 2	Headache	Severe
Case 6	Headache	Mild
	Nausea, vomiting	Moderate
Case7	Headache	Severe
Case 9	Vertigo, lightheadedness	Severe
	Nausea, vomiting	Severe
Case 16	Headache	Moderate
	Vertigo, lightheadedness	Severe
Case 22	Headache	Severe
Case 23	Headache	Severe

**Table 2:** Demographic characteristics of the studied groups

		Mean
Age (yrs)		58.10 ±10.89
Female to male ratio		16/4
Height (cm)		166.85±6.69
Weight (kg)		80.45±12.09
BMI (kg/m2)		29.21±4.21
Diabetes	Type 1 (%)	16 (53.3)
	Type 2 (%)	14 (46.7)
Duration of diabetes (yrs)		14.76±6.32
Duration of diabetic neuropathy (yrs)		3.06±2.94

Note: No difference in the demographics between the two groups since the patients had the same treatments, i.e. a crossover study

**Fig. 2:**
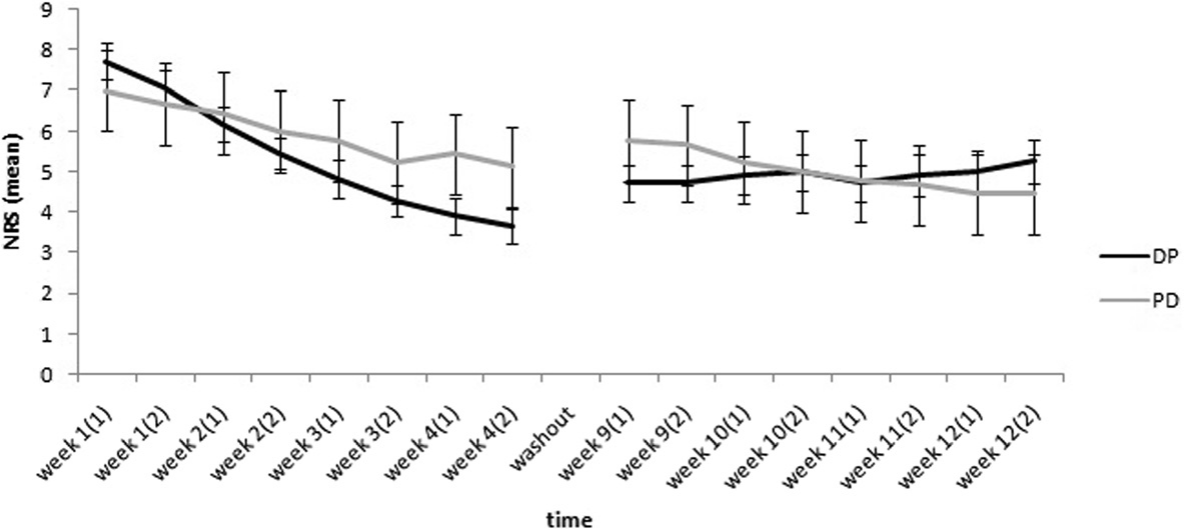
Pain intensity alterations, measured twice weekly (numbers in parentheses) during both phases of the study. At the end of the forth week the drug treatment were switched between the two groups

Studying NRS values in different intervals, using repeated measure ANOVA test, showed more reduction in mean pain scores in drug compared to placebo phase mainly during the pre-washout period of the study (*p* = 0.045 and 0.048 for the last two measurements of the forth week, respectively) (Fig. 2). The mean values were not statistically different during the post-washout phase. The consumption of the drug was associated with a 50 % reduction in the severity of the pain in 70 % of the patients. Such a pain relief however was only noted in 5 % of the patients in the placebo group. The changes noted in the severity of pain and allodynia are noted in Table 3. The pure effect of drug compared to placebo group in the severity of allodynia and other pain qualities was statistically significant (*P*-value < 0.001). Similarly, except for mood and sleep, a significant reduction in all Brief Pain Inventory scores was noted in the Drug group (*P*-value < 0.001) (Table 4). SF-MPQ also showed that using transdermal nitroglycerin is associated with a significant improvement in different aspects of pain measured using McGill Pain Questionnaire, not including Role–emotional (*P*-value < 0.001) (Table 5).

**Table 3:** Number of patients presented with different pain characteristics during the two phases of the study

		Asymptomatic	Mild	Moderate	Severe	*P* value*
Allodynia	Baseline	3	11	5	1	<0.001
	After drug treatment course	3	9	8	0	
	After placebo treatment course	0	3	14	3	
Other pain characteristics	Baseline	2	11	6	1	<0.001
	After drug treatment course	2	10	6	2	
	After placebo treatment course	0	2	14	4	

*Between group values analysed by Chi-Square Test

There is no difference in the baseline pain characteristics between the two groups since it is a crossover study

**Table 4:** Brief pain inventory score in the studied groups [Median (IQR)]

	Baseline	After placebo treatment	After drug treatment	Placebo difference	Drug difference	*P*-value*
Worst pain 24h	8(3)	7(3)	5(3.75)	-1.5(1)	-3.5(2.75)	0.004
Least pain 24h	4(2.75)	4(2)	2.5(2)	-1(1)	-2(2)	<0.001
Average pain 24h	6(2)	5.5(2)	4(2.5)	-1(2)	-2(1)	<0.001
General activity	6(1)	5(2)	3(2.75)	-1(2)	-3(1)	<0.001
Mood	5(2)	4(1.75)	3(2.75)	-1(0)	-1.5(2.75)	0.058
Walking	5.5(2)	5(3.5)	4(2.75)	-1(1)	-1.5(1.75)	0.016
Normal work	5(1)	4(1.75)	3(1.75)	-1(2)	-2(.75)	0.017
Social relations	5(2)	4(1)	3(1)	-1(.75)	-2(2)	0.042
Sleep	6(2)	4(2)	2.5(3.75)	-1.5(.5)	-3(1.75)	0.065
Enjoyment of life	5(2)	4(1.75)	3(2)	-1(1)	-2(1.75)	0.048

Data are presented as median (interquartile range)

^*^ *P*-value for drug difference and placebo difference according to Wilcoxon Signed Rank test

There is no difference in the baseline values between the two groups since it is a crossover study

**Table 5:** Pain measured using SF-MPQ in the studied groups

	Baseline median (IQR)	Placebo median (IQR)	Drug median (IQR)	Placebo pain difference median (IQR)	Drug pain difference median (IQR)	*P*-value*
Physical function (PF)	20(10)	25(13.75)	35(5)	5(10)	15(18.75)	.018
Role Functioning (RF)	25(25)	25(50)	50(0)	0(25)	25(53.75)	.026
Bodily pain (BP)	31(10)	41(16.50)	62(18.50)	11(10.50)	30.5(19.25)	.001
General health (GH)	20(12)	35(28.75)	53.50(32.50)	10(19.50)	29(16)	.001
Vitality (V)	27.50(8.75)	35(23.75)	55(28.75)	10(15)	25(8.75)	.002
Social functioning (SF)	37.50(25)	50(9.37)	75(21.87)	12.50(12.50)	25(7.50)	.005
Role-emotional (RE)	33.30(33.30)	66.70(33.40)	33.30(33.40)	33.30(33.40)	16.65(33.30)	.333
Mental health (ME)	32(8)	42(18)	58(31)	8(9.50)	24(22.50)	.002
Physical component summary (PCS)	26.05(5.05)	29.80(8.6)	38.35(11.85)	3.10(6.47)	11.45(12.27)	.001
Mental component summary (MCS)	31.25(7.47)	39.70(8.9)	43.50(10.65)	5.55(5.77)	11.10(6.67)	.012

Data are presented as median (interquartile range)

^*^ *P*-value for drug difference and placebo difference according to Wilcoxon Signed Rank test

There is no difference in the baseline values between the two groups since it is a crossover study

Using Beck Depression Inventory score system, the drug was reported to be effective in relieving depression associated with diabetic neuropathy (11.5 ± 8.03 vs 13.5 ± 7.02; *p* value = 0.006). The standard seven-point Patients’ Global Impression of Change (PGIC) scale showed that 80 % (95 % CI: 56 %–94 %) of the patients were satisfied with using the drug. As for the placebo group, the rate was as low as 20 % (95 % CI: 5.7 %–43 %) (*p* value < 0.001).

Except for those who left the study, no severe adverse effects were noted in the subjects. This comes while three of the patients experienced mild headache and two others had skin rashes at the site where the plasters were attached. None however needed any further treatments. None of the subjects reported the use of tramadol at any stage of the study.

## Discussion

The etiological factors attributed to DPN can be grouped into those having a definite role (e.g. poor glycemic control, duration of disease) and those with a probable added influence (e.g. hypertension, age, smoking, hyperinsulinemia, dyslipidemia) [19, 20]. Good glycemic control delays or prevents the onset of diabetic neuropathy and ameliorates symptoms in those with acute painful neuropathy [21]. However, even excellent glycemic control may be insufficient in some patients.

In the absence of curative therapy, the main aim of management is to provide symptomatic pain control using pharmacological and non-pharmacological agents, and to preserve good glycemic control [21]. The best reported results in controlling DPN have been obtained with antidepressants, reporting the drug to have a significant dose-dependent effects in reducing burning, aching, sharp, throbbing, and stinging pain in diabetic patients [8, 9]. This comes while major issues such as whether the pain-relieving effect is a result of decreased depression, or its serotonergic effects, noradrenergic properties or direct analgesic effect, have been raised with regard to the pain relief from antidepressants [22]. The drug however cannot be used in all patients because of its frequent side effects.

As for the anticonvulsants, the use of phenytoin and carbamazepine has shown promising results. Phenytoin however is problematic in diabetics due to its inhibitory effects on insulin secretion and long-term carbamazepine may cause serious hematologic side effects [23, 24]. The use of sodium valproate on the other hand is well-tolerated, and associated with significant subjective improvement in painful diabetic neuropathy. Unlike other anti-epileptic drugs, sodium valproate has a favorable side effect profile [25].

Gabapentin is widely used to treat DPN as despite being effective in reducing pain it has fewer troublesome side effects and almost no drug interactions [26, 27]. Unlike gabapentin, pregabalin exhibits linear pharmacokinetics across its therapeutic dose range, with low intersubject variability [28]. Pregabalin is also well tolerated despite a greater incidence of dizziness and somnolence [29].

Nowadays, tramadol, a centrally acting, synthetic, non-narcotic analgesic, is commonly used for the treatment of moderate to moderately severe pain, even chronic pain in diabetic neuropathy [30]. Despite its low potential for abuse, tramadol should not be used in opioid-dependent patients or those with a tendency to abuse drugs.

Antagonists of the N-methyl-D-aspartate (NMDA) glutamate receptor such as Dextromethorphan are also reported to be effective in reducing pain of diabetic neuropathy but not postherpetic neuralgia [31]. Duloxetine was also considered to be safe and well tolerated with less than 20% discontinuation due to adverse events [32, 33].

Recently, Yuen et al. for the first time used isosorbide dinitrate (ISDN) spray to reduce pain in DPN subjects [17]. They reported that the improvement in pain and burning sensation noted in these patients may be associated with the increased generation of NO, promoting vasodilation with secondary improvement in microvascular blood flow. In other words, they reported that the vasodilation induced by the increased generation of NO following ISDN treatment may induce angiogenesis of the vasa nervorum, causing the gradual increase noted in the analgesic effect of the spray by the end of the first week. Alternatively, the ISDN spray may have stimulated the light-touch peripheral receptors of the A fibers, thus suppressing neuropathic pain. According to the gate-control theory, the activity generated by myelinated primary afferent fibers (A fibers) blocks the transmission of activity in the small unmyelinated C fibers [34].

In the present study transdermal nitroglycerin patches were used to combine the effectiveness of nitroglycerin as reported in the Yuen study and transdermal patches as noted by Rayman. Our study showed that these patches reduced pain during the pre-washout period. After this washout interval the difference was not significant (maybe due to loss of about one third of the participants mainly because of drug side effects). At the same time most patients experienced some general improvement and well-being. While our study failed to assess different types of pain separately, we reported that patients experience more pain of any type while using the placebo. Moreover, our study revealed that nitroglycerin is more effective in reducing physical pain than improving the mental status of the patients, pointing out the complicated nature of chronic pain and need for multi-aspect treatment of the condition [35].

The main limitation to the use of these plasters is the high rate of side effects experienced by the consumers, a condition which made a large number of our subjects leave the study and may overestimate the obtained results. This is also in line with the result of previous studies which have reported headache in 52 % of nitroglycerin users.

## Conclusions

It could be concluded that nitroglycerin plasters can effectively help alleviate pain in patients with diabetic neuropathy. Further studies with larger patient numbers are required to confirm the findings of this study, and determine whether the effects are sustained. Moreover considering the promising results of this study, the effect of these plasters on diabetes status is an issue of concern and needs to be studied in the future.

## Abbreviations

BDI: Beck depression inventory
BPI: Brief pain inventory
DPN: Diabetic peripheral neuropathy
GTN: Glyceryltrinitrate
ISDN: Isosorbide dinitrate
NRS: Numerical rating scale
PGIC: Patients’ global Impression of change
SF-MPQ: Short-form- McGill pain questionnaire
